# 50,000 years of ice and seals: Impacts of the Last Glacial Maximum on Antarctic fur seals

**DOI:** 10.1002/ece3.8104

**Published:** 2021-09-10

**Authors:** Alison C. Cleary, Joseph I. Hoffman, Jaume Forcada, Christian Lydersen, Andrew D. Lowther, Kit M. Kovacs

**Affiliations:** ^1^ Department of Natural Sciences University of Agder Kristiansand Norway; ^2^ Norwegian Polar Institute Fram Centre Tromsø Norway; ^3^ Department of Animal Behaviour University of Bielefeld Bielefeld Germany; ^4^ British Antarctic Survey Cambridge UK

**Keywords:** *Arctocephalus gazella*, demographic history, glacial refugia, single nucleotide polymorphisms

## Abstract

Ice is one of the most important drivers of population dynamics in polar organisms, influencing the locations, sizes, and connectivity of populations. Antarctic fur seals, *Arctocephalus gazella*, are particularly interesting in this regard, as they are concomitantly reliant on both ice‐associated prey and ice‐free coastal breeding areas. We reconstructed the history of this species through the Last Glacial Maximum (LGM) using genomic sequence data from seals across their range. Population size trends and divergence events were investigated using continuous‐time size estimation analysis and divergence time estimation models. The combined results indicated that a panmictic population present prior to the LGM split into two small refugial populations during peak ice extent. Following ice decline, the western refugial population founded colonies at the South Shetlands, South Georgia, and Bouvetøya, while the eastern refugial population founded the colony on Iles Kerguelen. Postglacial population divergence times closely match geological estimates of when these coastal breeding areas became ice free. Given the predictions regarding continued future warming in polar oceans, these responses of Antarctic fur seals to past climate variation suggest it may be worthwhile giving conservation consideration to potential future breeding locations, such as areas further south along the Antarctic Peninsula, in addition to present colony areas.

## Graphical abstract

We reconstructed the history of Antarctic fur seals through the Last Glacial Maximum (LGM) using genomic sequence data from seals across their range. Our results indicated that during the LGM, the global population was reduced to two small refugial populations, and that these populations then expanded shortly after the ice retreat to found the present‐day colonies.

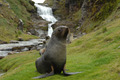

## 1 INTRODUCTION

Ice is a key environmental driver for many polar organisms, with glaciers, snow, and sea ice often determining the size and connectivity of populations through diverse mechanisms. Ice can influence populations directly by creating or restricting access to potential habitat areas (Kovacs et al., 2011; Massom & Stammerjohn, 2010; Siniff et al., 2008; Younger et al., 2016). Ice can also influence populations indirectly through trophic links. For example, changes in sea ice algae can drive bottom‐up ecosystem effects, while changes in the availability of sea ice as a hunting platform for organisms such as leopard seals and polar bears can result in top‐down effects (Kovacs et al., 2011; Reid et al., 2005; Siniff et al., 2008).

Understanding how species were historically impacted by changing environmental conditions and loss of appropriate habitat areas may provide information relevant to conservation planning for the future. During the LGM, approximately 25–15 thousand years ago, glaciers covered most of the Antarctic continent and the sub‐Antarctic islands, seasonal sea ice extended to 45°S, and primary productivity was reduced (Allcock & Strugnell, 2012; Fraser et al., 2012; Hall, 2004; Mortlock et al., 1991). These changes in ice had diverse impacts across species, including changes in geographic distributions (range shifts, contractions, and expansions), changes in population structure (merging or splitting of populations), and changes in population size (increases and decreases), with some species showing very large effects, while others were less impacted (Allcock & Strugnell, 2012; Younger et al., 2016).

The Antarctic fur seal, *Arctocephalus gazella*, is an abundant pinniped in the Antarctic and sub‐Antarctic that serves as an ecosystem indicator for Southern Ocean management (CCAMLR, 2014; Reid et al., 2005). *A*. *gazella* breeds on sub‐Antarctic islands around the continent with very high site fidelity (Hoffman et al., 2006) and forages across broad areas of the Southern Ocean outside of the breeding season (Forcada & Staniland, 2018). Virtually, nothing is currently known about how this species responded to the LGM. Contemporary observations (over the last 30 years), and paleontological studies (covering the last 1,500 years), have both suggested that sea ice variations impact *A*. *gazella* populations, both directly and indirectly (Siniff et al., 2008; Sun et al., 2004; Waluda et al., 2010). *A*. *gazella* populations are directly affected by ice as they need ice‐free coastal areas for pupping and rearing offspring (Forcada & Staniland, 2018). Indirectly, ice impacts *A*. *gazella* through both bottom‐up effects on their main prey, the Antarctic krill *Euphausia superba*, and via top‐down effects on their principal predator, the leopard seal *Hydrurga leptonyx*, both of which are considered ice‐dependent species (Schwarz et al., 2013; Siniff et al., 2008).

**FIGURE 1:**
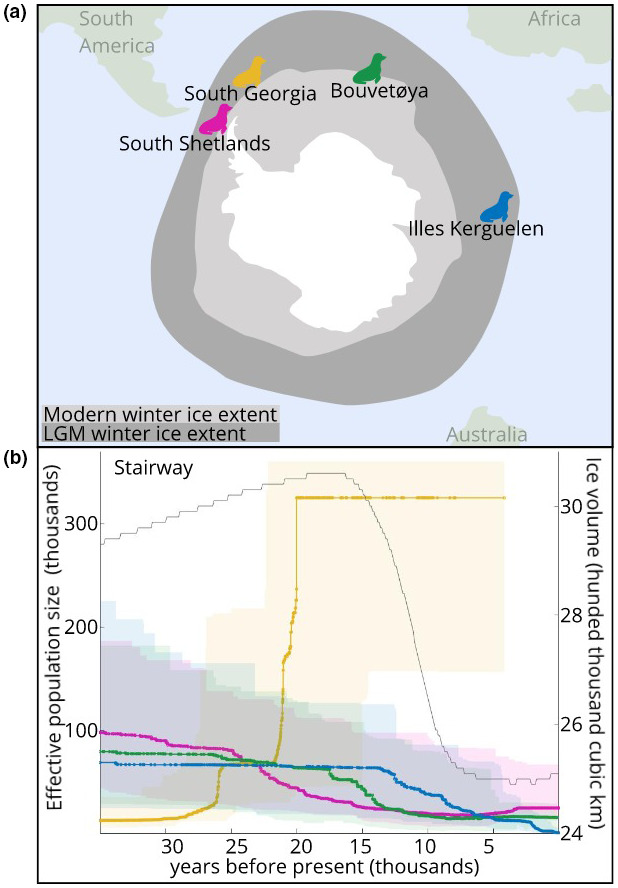
Sample locations and effective population size estimates. (a) Map of colony locations and winter sea ice extent (present and LGM). (b) Effective population size changes over time for each colony. Connected points indicate median values, and shaded areas indicate the corresponding 95% confidence intervals from 200 bootstrap replicates. The results for each island are also available in separate panels in the Supplemental material. Ice extents re‐drawn from Allcock and Strugnell (2012), ice volume data (gray lines in b) from De Boer et al. (2014)

We aimed to determine how population size and structure of *A*. *gazella* changed during and following the LGM, to better understand the responses of this species to large‐scale climate change. We analyzed genomic data from 52 seals from four present‐day colonies (the South Shetlands, South Georgia, Bouvetøya, and Iles Kerguelen) (Figure 1). Effective population sizes were estimated continuously across time for each colony, and a series of models were used to estimate divergence order and divergence times among these four populations. These analyses combine to provide a new picture of the history of this species across a period of dramatic climate change.

## 2 MATERIALS AND METHODS

### 2.1 Sampling

Blood or skin samples were collected from 13 *A*. *gazella* individuals on each of the following islands: Livingston Island in the South Shetlands (60.82°W 62.61°S), Bird Island on South Georgia (38.05°W, 54.01°S), Bouvetøya (3.35°W 54.42°S), and Iles Kerguelen (69.39°E 49.36°S). All sampling was performed under permits: South Shetland Islands—US National Marine Fisheries Service, Marine Mammal Protection Act permit #774‐1847‐04; South Georgia—British Antarctic Survey Animal Welfare and Ethics Review Body permit #PEA6; Bouvetøya—Norwegian Department of Plants, Fish, Animals and Food permit #7001, and Iles Kerguelen—CNRS‐Chizé permit. Samples were preserved in ethanol or sodium chloride saturated dimethyl sulfide, and/or frozen until processing.

### 2.2 DNA sequencing

DNA was extracted using a chloroform‐isoamyl alcohol protocol modified from Sambrook et al. (1989) as in Paijmans et al. (2020). Double digestion restriction‐associated DNA sequencing library preparation, consisting of digestion with the enzymes SphI and EcoRI, and ligation of dual, variable length, individual identification tags, and sequencing adaptors, was conducted by IGA Technology (Peterson et al., 2012). The resulting libraries were then paired‐end sequenced on an Illumina HiSeq 2500.

### 2.3 Site frequency spectra

Quality control and de‐multiplexing were conducted with process_radtags in Stacks using default parameters (Catchen et al., 2013). Sequences were mapped against an *A*. *gazella* reference genome (NCBI accession #SRP148937) using Bowtie2 (Langmead & Salzberg, 2012). Sequences were reformatted as a sorted BAM file in SamTools (Li et al., 2009). Genotype likelihoods were calculated for all base positions within mapped reads using the Genome Analysis Tool Kit (GATK) model in Analysis of Next Generation Sequencing Data (ANGSD), with a SNP *p*‐value cutoff of 10^–6^, a quality threshold of 20, and a minimum sequencing depth of 2 (Korneliussen et al., 2014; McKenna et al., 2010). Genotype likelihood‐based approaches take into consideration the certainty of each SNP, which allows for even low‐coverage sites to contribute to the analyses (Korneliussen et al., 2014). Most of the analyzed SNPs were covered to much greater depth, with an average sequencing depth for analyzed SNPs of 24.98 across the entire dataset (calculated excluding sites present in excess of 100×). Sequences mapping to more than one location in the genome, or with a mapping quality of less than 1, were excluded from analyses. Fst values were calculated to provide a basic measure of neutral, genome‐wide differentiation (see Table S1).

Site frequency spectra (SFS) for each colony alone, for all possible pairs of colonies, and for all colonies together were calculated from the genotype likelihoods in RealSFS, as implemented in ANGSD (Korneliussen et al., 2014; Nielsen et al., 2012). Additionally, SFS were calculated for two pairs of artificial “populations,” which were composed of in silico mixes of randomly selected individuals from the South Shetlands and South Georgia, to be used as a control group. SFS calculations assumed Hardy–Weinberg Equilibrium, which is reasonable for this data, as Cleary et al. (2019) showed that the vast majority of the SNPs were in HWE in the study populations. Single‐colony SFS were calculated as folded, while multicolony SFS were calculated as unfolded, and subsequently folded in Moments (Jouganous et al., 2017), as the format of multicolony folding differs between these two packages. All size bins were retained in the SFS (i.e., singletons were not discarded). Including singletons has been shown to provide the most accurate SFS when using a likelihood‐based approach such as ANGSD, unlike when working with called SNPs where singleton counts are often inflated due to calling errors (Han et al., 2014; Nielsen et al., 2012). Each SFS was based on over 15 million DNA sequence positions (see Table S2 for exact counts).

### 2.4 Individual colony size estimation over time

All modeling assumed a mutation rate of 1.2 * 10^–8^ mutations generation^−1^, a value well validated in humans, and commonly applied to nonmodel mammals (Liu & Fu, 2015; Trost et al., 2021; Wiens, 2021), and a generation time of 10 years (Paijmans et al., 2020) (see Supplemental material for the effects of varying these parameters). To ensure unbiased conclusions, only total Antarctic ice volume was included in intermediate visualizations during analyses; regional variations in ice timing and extent were not investigated until the final models were selected based on the genetic data.

Population sizes were estimated continuously through time for each of the four colonies using Stairway2 (Liu & Fu, 2020). Stairway estimates effective population size independently for each time interval; it has been validated with simulated populations as small as 15 diploid individuals (Liu & Fu, 2015, 2020). The percentage of sites used in training the model followed the author's recommendation of 67%. Random break points were 6, 12, 20, and 24 (again following the author's recommendations—here spaced over the sample size of 26 haploid genomes). Each estimation was based on 200 bootstraps. Complementary analyses using additional scenario‐free approaches were attempted to increase overall confidence, but as these led to either extremely wide confidence intervals (Epos—Lynch et al., 2020) or over‐smoothed curves (CubSFS—Waltoft & Hobolth, 2018), they were not pursued further (see Supplemental material).

### 2.5 Population divergence time estimation

Population divergence times were estimated in Moments (Jouganous et al., 2017). This approach was chosen because it is computationally efficient and more clearly documented than the other available packages. We aimed for a model that was as simple as possible, while still providing meaningful information on population divergence times. The more parameters a model contains, the greater the risk of over‐fitting, and the more computationally unwieldy it becomes. Therefore, preliminary models, including two islands at a time, were used initially to generate a scaffold of the likely population divergence history, and then, models were built including all four populations based upon those results.

In order to test a very broad range of parameters and to maximize resolution by increasing runs near the optimal values, parameter ranges were iteratively tightened for all models. Very broad parameters were run initially, with divergence times ranging from 1 to 100,000 generations. These initial broad runs minimized the risk of converging to a local maximum, as each run begins from a random set of parameter values within this broad space, and then optimizes from that point. Results were analyzed every 200–500 runs, and subsequent runs were started with parameter values encompassing those observed in the highest likelihood 10% of the results obtained up to that point. In all models, each population size was free to vary compared to other populations, but each was fixed across time. Population size changes over time were not included into the moments models, as this would have introduced many additional parameters (e.g., adding an LGM bottleneck would require a start time, an end time, and a bottleneck size for each refugial population). The size of each population, though fixed across time, was free to vary widely (from roughly 2 to 2 million individuals). This should allow the model to select population sizes that are broadly representative across the study period, and minimize impacts of this simplification on the estimated divergence times.

#### 2.5.1 Two‐population models

Two‐population models simulated a single ancestral population splitting into two populations. These models include two parameters to optimize—the time of the split (in generations prior to the present), and the ratio of sizes between the two populations (total population size is estimated in all models as it is used as a scaling factor for both size and time). All possible pairs of islands, as well as the two control pairs, were run in two‐population models for 500 iterations. The timing of very recent divergence events can be challenging to estimate, as there has been little opportunity for the populations to accumulate differences. The control pairs were used to evaluate what divergence times would be generated from populations that are not truly diverged, in order to set an informal lower confidence limit on recent divergence events. Lastly, we also tested all possible pairs of islands using a slightly more complex two‐population model that included continuous symmetric migration (0–30 coalescent units) from the point the populations split to the present.

#### 2.5.2 Four‐population models

Two four‐island models were built based on the results of the two‐population models. In both models, the divergence order was fixed, but as with the two‐population models, the size of each population and the time of each divergence event were allowed to vary.

In the bifurcating model, the order of divergence was as follows: first Iles Kerguelen, then Bouvetøya, and lastly the South Shetlands and South Georgia. This model allows for one to four refugial populations during the LGM: 1—a single refugial population, with all divergence events subsequent to ice retreat; 2—a western refuge (South Shetlands, South Georgia, and Bouvetøya) and an eastern refuge (Iles Kerguelen); 3—only South Georgia and the South Shetlands having a shared refugial population; or 4—each island belonged to a separate refugial population, with all divergence events occurring prior to the LGM. This model was run 1,500 times to estimate the most likely time for each of the three divergence events.

Finally, we evaluated the star‐like model, which simplified the bifurcating model slightly, by having the South Shetlands, South Georgia, and Bouvetøya all diverge simultaneously. This star‐like model reduces the number of free parameters from seven in the bifurcating model to six. This star‐like model was run 2,500 times. With a calculation time of roughly 5 cpu hours per run for the four‐population models, the results of these two models represent approximately 12,500 cpu hours (see Supplemental material for model convergence metrics).

## 3 RESULTS

### 3.1 Effective population size over time

Estimated effective population size declined around the period of the LGM at the South Shetlands, Bouvetøya, and Iles Kerguelen (Figure 1). These reductions occurred sequentially, with South Shetlands declining first, followed by Bouvetøya, and lastly Iles Kerguelen. By contrast, the South Georgia population showed an increase around this time period.

### 3.2 Population divergence times

#### 3.2.1 Two‐population models

**FIGURE 2:**
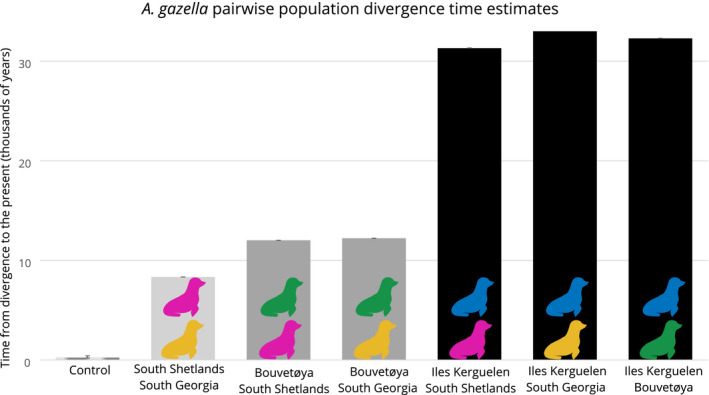
Population split timings estimated for all possible pairs of islands. Seal icon colors (as in Figure 1), and *X*‐axis labels indicate the pair of islands whose divergence time is estimated in each bar. One of the control models is included on the far left. Error bars show range within the 50 highest likelihood models (error bar size ranges from 1 to 197 years). Bar shading highlights how these preliminary models were used to inform bifurcation order in the four‐population models. The light gray bar shows the most recent divergence event (the South Shetlands and South Georgia), and medium gray bars show the intermediate branching island (Bouvetøya) when paired with either of these two islands; black bars show the earliest branching island (Iles Kerguelen), when paired with all other islands (note that the control bar, in silico populations consisting of randomly selected individuals from the South Shetlands and South Georgia, is so small it is barely visible at this scale)

The two‐island models indicated a clear branching order—the South Shetlands and South Georgia diverged from one another most recently, Bouvetøya diverged from both the South Shetlands and South Georgia at an intermediate timepoint, while Iles Kerguelen diverged much earlier from all of the other islands (Figure 2). These patterns were consistent across model runs; only within the lowest likelihood 2% of the model runs are there observed any results which could suggest a different branching order.

The control models estimated population divergence times that were much more recent than the South Shetlands and South Georgia paired island model, as expected for such in silico mix populations (random mixes 1 and 2: best fit 223 years, range in top 50 models 202–420; random mixes 3 and 4: best fit 701, range in top 50 models 700–701, actual South Georgia and South Georgia best fit 8,337, range in top 50 models 8,336–8,338) (Figure 2). This increased our confidence that even the most recent divergence time estimates represented meaningful inference, and not stochastic effects or methodological artifacts.

Including migration had very little effect on the model fits (Figure S4). For the island pairs that included Iles Kerguelen, models with migration gave very similar split timings to models without migration (population split timing estimated by best fit models: SS_KI with migration 31, 423, without migration 31, 315; SG_KI with migration 34,266, without migration 34,365; BI_KI with migration 32,295 without migration 32,297). The models of island pairs with more recent divergence times (SS_SG, SS_BI, and SG_BI) were unstable when migration was included, with large variability between the highest likelihood runs. Based on these results, we did not incorporate migration into the four‐population models.

#### 3.2.2 Four‐population models

In the first of our four‐population models (bifurcating), each island split off at a separate time point, first Iles Kerguelen, then Bouvetøya, and lastly the South Shetlands and South Georgia. In the second four‐population model (star‐like), Iles Kerguelen split off first, followed by the simultaneous divergence of Bouvetøya, the South Shetlands, and South Georgia.

**FIGURE 3:**
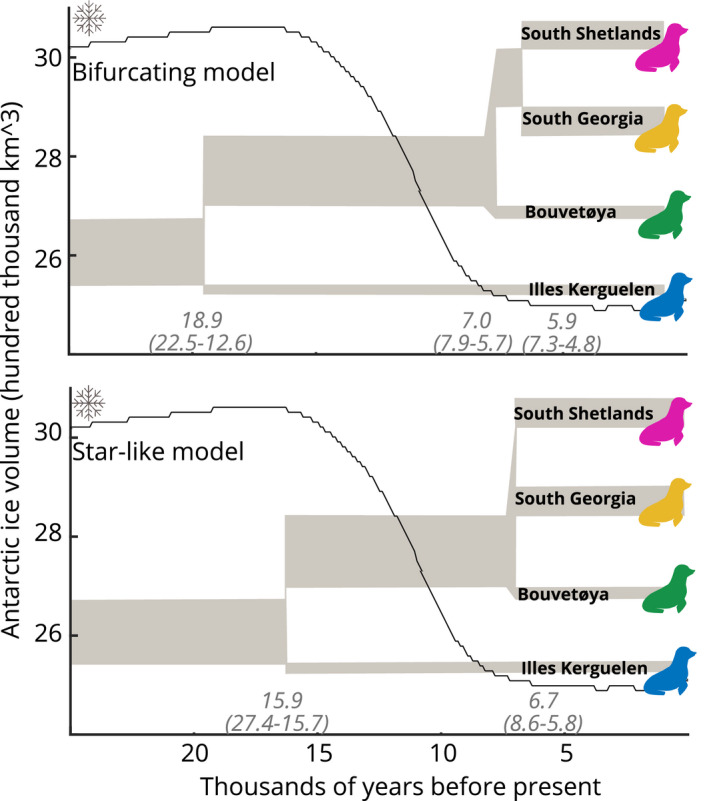
Best‐fit population divergence models. Population size (relative to the ancestral predivergence population) and divisions are shown in gray shading, and split times (range of the top ten model fits) are indicated above the *x*‐axis in italics. Ice volume is shown as a black curve with a snowflake (De Boer et al., 2014)

Both of the four‐island divergence time models indicated that Iles Kerguelen diverged from the other populations around the peak of LGM ice volume (best fit bifurcating: 18,948 years ago, range in highest likelihood 10 models: 22,494–12,650, best fit star‐like: 15,884, top 10: 27,443–15,724) (Figure 3). Both also indicated that the other three colonies diverged after the ice retreated, with the bifurcating model indicating Bouvetøya diverged from the others 7,033 years ago (top 10: 7,952–5,748), with the South Shetlands and South Georgia diverging shortly thereafter at 5,958 years ago (top 10: 7,352–4,826), while the star‐like model estimates a divergence time for all three intermediate to these, at 6,749 years ago (top 10: 8,650–5,774). The bifurcating model resulted in a slightly higher likelihood, but the difference was small compared to inter‐run variation (see Supplemental material).

## 4 DISCUSSION

### 4.1 Methodological considerations

The divergence order indicated by two of the population models is consistent with the population structure indicated by Fst values (see Supplemental material) as well as with the results of previous studies (Cleary et al., 2019; Paijmans et al., 2020; Wynen et al., 2000).

Overall, the results of our analyses were highly consistent, but there was a single exception. Divergence time models estimate that South Georgia, the South Shetlands, and Bouvetøya were a single population before about 7 kya, yet the Stairway analysis suggested that South Georgia followed a different trajectory as far back as 20 kya. Lapierre et al. (2017) showed that simple scenario‐based models, such as the divergence time models used here, often provide more accurate representations of a populations' demographic history than scenario‐free approaches, such as Stairway plots, particularly when working with limited data. The incongruous growth trajectory in the Stairway results for South Georgia may be an artifact of applying a model optimized for relatively gradual population change to a population that increased very rapidly in the recent past. Surveys indicate that the South Georgia population expanded from only a handful of individuals in the 1920s to 4–6 million today (Forcada & Staniland, 2018). We therefore consider that in this one point where the Stairway results disagree with the divergence time models, the divergence time models likely provide the most accurate inference.

Migration is notoriously challenging to include in demographic models based on genomic data (Jouganous et al., 2017). Here, we addressed the question of migration in our simplest, two‐population models. The finding that there was no difference in divergence times when including versus ignoring migration in models including the deepest branching Iles Kerguelen indicates that migration between distant islands has historically not been an important factor. Results for the more closely related and geographically proximate islands were more ambiguous; these models failed to converge when migration was included, which could potentially reflect some low‐level historical migration (either symmetric or asymmetric) but could also simply reflect that more recently diverged populations are more challenging to model. Low level unaccounted for migration could potentially impact our findings in two ways. Immigration into South Georgia from the South Shetlands, Bouvetøya, or a now extinct ghost population, could be an additional explanation for the anomalously high population size estimates Stairway produced for South Georgia. Secondly, unaccounted for migration between the three most recently diverged islands could have led to an underestimation of the time since these islands diverged. However, there is very little migration between colonies of this species at present, as evidenced by the strong population structure (Cleary et al., 2019), very high site fidelity in adults (Hoffman et al., 2006), and very low dispersal of novel phenotypes (Hoffman et al., 2018). Given this, and the two population model results, it seems unlikely that sufficient migration was present in the past to significantly shift our estimated divergence times, but this is an area, which could benefit from further research, particularly as both sequence data availability and analytical methods are likely to improve in future.

The two four‐population divergence time models performed similarly well in terms of the likelihood of each model's fit. Thus, we consider the simpler star‐like model, which contains one fewer fitted parameter, to be the most parsimonious explanation for *A*. *gazella* colony divergence events. We therefore focus on this model in the remainder of the discussion but note that the main conclusions would be the same with either model. Discerning the timing of these divergences with enough precision to confidently distinguish between a true star divergence and two bifurcations close in time would likely require more complete sequence data coverage.

### 4.2 Probable history of the species—two refugial populations

Combining all of our results, we consider the most probable history of this species to be as follows. Prior to the LGM, a population of Antarctic fur seals existed in the Antarctic and/or sub‐Antarctic. As the ice extent increased, much of this area became uninhabitable, and the global population was reduced to two small refugial subpopulations, one in the west and one in the east. Refugial areas would have needed ice‐free breeding habitat and productive foraging grounds, with potential locations north of present breeding areas or in areas with geothermal activity. After the ice declined, the western refugial population founded colonies at the South Shetlands, South Georgia, and Bouvetøya, while the eastern refugial population colonized Iles Kerguelen.

### 4.3 Alternative potential histories

#### 4.3.1 Alternative potential history—one refugial population

We cannot rule out a scenario with a single refugial population. The latest estimated divergence time for Iles Kerguelen from the top 10 highest likelihood fits of the bifurcating model (12,650 years ago) is around the time of the earliest geological estimates for when Iles Kerguelen had ice‐free coastal habitat (13,000 years ago) (Hodgson et al., 2014). However, the other nine of the highest likelihood bifurcating models, and all of the top 10 highest likelihood star‐like models, estimate this divergence earlier than 15,700 years ago, at a time when ice would likely have been extensive on and around Iles Kerguelen. Thus, a single LGM refuge history appears less likely than the two refugial population history.

#### 4.3.2 Alternative potential history—more than two refugial populations

It is also possible that there were more than two refugial populations. Additional refugial populations could have existed in the Western region, if unaccounted for migration led the modeling results to significantly underestimate divergence times. However, as discussed above, this scenario appears unlikely.

A more likely source of additional refugial populations lies with populations that were extirpated during 18th‐century commercial harvests. Harvest pressure was intense but short lived, so the impact on overall genetic diversity was small (Basberg & Headland, 2008; Bonner, 1968; Forcada & Staniland, 2018; Paijmans et al., 2020). However, some colonies, such as those of the Prince Edward Islands, Iles Crozet, Heard Island, and Macquarie Island, are thought to have been completely extirpated, with present‐day colonies at these locations founded within the last century (Cleary et al., 2019; Paijmans et al., 2020; Wynen et al., 2000). These colonies are thus now composed entirely of seals whose ancestors at the time of the LGM belonged to other colonies (hence their exclusion from the analyses presented here).

Based only on data from modern‐day individuals, it is impossible to know whether there may have been additional LGM refugial populations that left no remaining descendants, in places such as on ice‐free areas of Iles Crozet, on Macquarie Island or on small islands around New Zealand. Future work with museum specimens would be a possible avenue to detect such refugial populations, although this question may ultimately prove intractable as capture locations were often not recorded by sealers (Basberg & Headland, 2008).

### 4.4 Glaciological context

Our results, and the probable history they indicate for Antarctic fur seals with two refugial colonies, are consistent with the results of geological studies on the timing of ice expansion and retreat in different areas of the sub‐Antarctic. The LGM ice cover on both land and sea lasted longest at the most poleward locations, which experienced both earlier ice expansion and later retreat (Hodgson et al., 2014).

At the beginning of the LGM, the South Shetlands would have been the first area to become uninhabitable due to extensive ice, followed by South Georgia and Bouvetøya, with Iles Kerguelen being the last island to experience significant glaciation and coastal sea ice. This is consistent with the later population decline seen in the Stairway analysis for the eastern (Iles Kerguelen) refugial population as compared to the Western populations. At the end of the LGM, the opposite trend occurred. Geological studies show coastal areas were ice‐free much earlier at Iles Kerguelen (11–13 thousand years ago) than at South Georgia (6–7.5 thousand years ago), and the South Shetlands (5.2–6.2 thousand years ago) (Hodgson et al., 1998, 2014). No data are available for Bouvetøya (Hodgson et al., 2014), but its position near today's polar front suggests that it would have been intermediate to South Georgia and Iles Kerguelen. The earlier divergence of Bouvetøya in the bifurcating model may reflect earlier availability of breeding habitat on this island, as compared to the South Shetlands and South Georgia. This estimated timing of ice retreat on the South Shetlands and South Georgia fits closely the estimated divergence time for these populations from the genomic data (6–7 thousand years ago), suggesting *A*. *gazella* refugial populations colonized these islands and established new populations shortly after these habitat areas became available.

### 4.5 Comparison with other marine mammals

While relatively little is known about the responses of marine mammals to the LGM, our results are consistent with the few existing studies. The demographic history of killer whales, *Orcinus orca*, as inferred from mitochondrial sequences, indicated a decline in populations during the LGM, with subsequent increases following ice retreat (Moura et al., 2014). This is similar to our observations from Stairway analyses, showing smaller populations of *A*. *gazella* during peak ice extent. Analysis of ancient remains of Southern elephant seals, *Mirounga leonina,* has indicated that they established a new breeding colony near the Ross Sea around the time this coastline became ice‐free following the LGM, 7,500–8,000 years ago (de Bruyn et al., 2009). Southern elephant seals share several ecological characteristics with *A*. *gazella*, including breeding on ice‐free beaches in the Antarctic and Sub‐Antarctic, and returning to the same breeding beaches year after year. The finding that both *M. leonina* and *A*. *gazella* established new breeding colonies quite close to the times that these locations became ice‐free following the LGM suggests that the potential to establish new colonies when new habitat becomes available may be common among highly mobile pinnipeds, even those species with generally high breeding site fidelity.

### 4.6 Implications for the future

Conservation concern for *A*. *gazella* has focused around the geographic locations of present‐day colonies, as this species exhibits strong site fidelity and seasonally restricted movement associated with rearing offspring (Forcada & Staniland, 2018; Hill et al., 2016; Hoffman et al., 2006). Our results, indicating persistence of this species in glacial refugia and subsequent founder events, adds to existing results on postharvest re‐colonization (Cleary et al., 2019; Paijmans et al., 2020), in showing that this species has the capacity to establish new breeding colonies when suitable habitat becomes available. Given the uncertainties around future climate change impacts, it may therefore be worthwhile giving conservation consideration to potential future breeding locations, such as areas further south along the Antarctic Peninsula, in addition to present colony areas.

## CONFLICT OF INTEREST

The authors have no conflicts of interest.

## AUTHOR CONTRIBUTIONS

**Alison C. Cleary:** Conceptualization (lead); formal analysis (lead); writing‐original draft (lead); writing‐review & editing (lead). **Joseph I. Hoffman:** Conceptualization (supporting); writing‐review & editing (equal). **Jaume Forcada:** Writing‐review & editing (supporting). **Christian Lydersen:** Funding acquisition (equal); writing‐review & editing (supporting). **Andrew D. Lowther:** Funding acquisition (supporting). **Kit M. Kovacs:** Conceptualization (supporting); funding acquisition (lead); writing‐review & editing (equal).

## Supporting information

Supplementary Material

## Data Availability

Original sequence data are available under bioproject RJNA521705 in the NCBI short read archive. All data processing and analysis scripts, as well as single‐colony SFS, are available in the supplemental material.
