# Supplementary material for: 50,000 years of ice and seals: Impacts of the Last Glacial Maximum on Antarctic fur seals
Source: Ecol Evol. 2021 Sep 10;11(20):14003–11. doi: 10.1002/ece3.8104 (PMC8525082; doi:10.1002/ece3.8104)
Supplement: Supplementary file 1 — Supplementary Material [file ECE3-11-14003-s001.pdf]

Supplementary material for:

50,000 years of ice and seals: impacts of the Last Glacial Maximum on Antarctic fur seals

Alison C Cleary, Joseph I Hoffman, Jaume Forcada, Andrew D Lowther, Christian Lydersen, Kit M Kovacs

Table of Contents

Supplementary tables 2
Table S1: Fst values for the analyzed islands 2
Table S2: Number of DNA sites included in each of the analyzed SFS 2
Table S3: effects of varying parameter values for generation time (gen) and mutation rate (mut) on estimated population divergence times (in years). 2
Supplementary figures 3
Figure S1: All demographic inference models – with each island and analysis method as a separate panel 3
Figure S2: Model fits for pairwise models with and without migration. 4
Figure S3: Model fit runs – loglikelihood, ... 4
Figure S4: Likelihood distribution for moments model runs 5
Figure S5: Model fit runs – population split timing – starlike model 5
Figure S6: Model fit runs – population split sizes 6
Scripts 7
Site Frequency Spectrum generation in ANGSD 7
Stairway 9
CubSFS 10
Epos 12
Moments 13
Scenario definitions 13
Model fitting 16
Model fitting shell 17
Illustration 18
Site Frequency spectra for single colonies 19

Supplementary tables

	LI	SG	BØ	IK
LI		0.092	0.108	0.172
SG			0.111	0.182
BØ				0.184
IK				

Table S1: Fst values for the analyzed islands Fst calculations were conducted in ANGSD, using SNP positions which were present in at least 4 individuals in each colony.

Island(s)	Total sites included in SFS
South Shetlands	25,790,417
South Georgia	24,157,307
Bouvetøya	23,185,013
Iles Kerguelen	31,037,895
South Shetlands-South Georgia	16,267,285
South Shetlands-Bouvetøya	16,186,781
South Shetlands-Iles Kerguelen	16,227,791
South Georgia-Bouvetøya	16,112,784
South Georgia-Iles Kerguelen	16,140,787
Bouvetøya-Iles Kerguelen	16,023,400
Four population	15,100,284

Table S2: Number of DNA sites included in each of the analyzed SFS

Division	gen: 10 years mut: $1.2*10^{-8}$	gen: 8 years mut: $1.2*10^{-8}$	gen: 10 years mut: $1*10^{-8}$	gen: 8 years mut: $1*10^{-8}$
SS-SG-BI	6749	8100	5400	6480
IK	15884	19061	12707	15249

Table S3: Effects of varying parameter values for generation time (gen) and mutation rate (mut) on estimated population divergence times (in years) for the starlike model. Varying the values used for generation time and/or mutation rate within reasonable bounds (from 10 to 8 years, and from 1.2*10-8 to 1*10-8, respectively) did not change the overall patterns of the timing of population divergences relative to the LGM ice volume peak The first column shows the results presented in the main article (generation time of 10 years and mutation rate of 1.2*10-8).

Supplementary figures

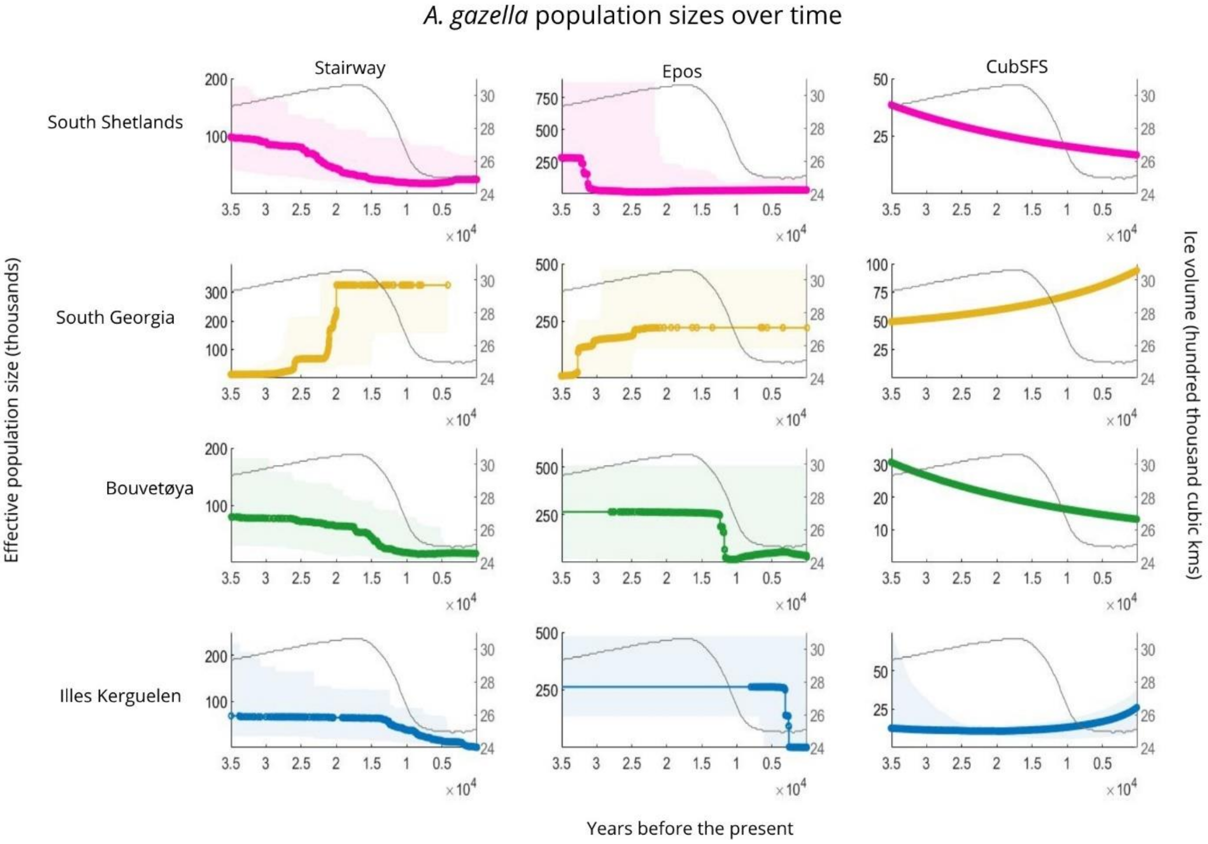

Figure S1: All demographic inference models – with each island and analysis method as a separate panel to allow for better visualization of confidence intervals. Connected points indicate median values, shaded areas indicate the corresponding 95% confidence intervals from bootstrap replicates (Stairway & CubSFS – 200, Epos – 2000). Note that each panel is plotted on a different y-axis scale, to maximize the detail visible, and that some narrow confidence intervals are hidden behind the median value points.

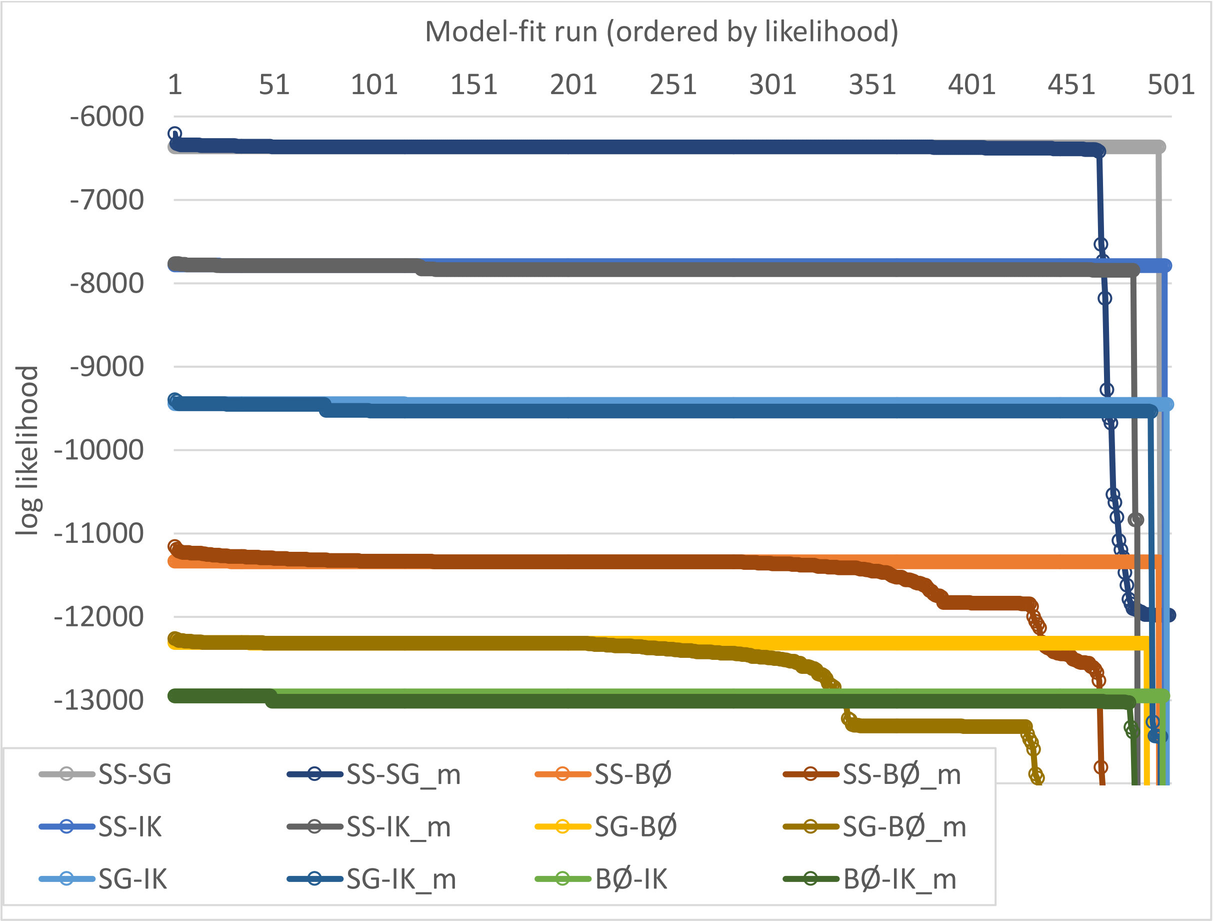

Figure S2: Model fits for pairwise models with and without migration. Note that migration does not have much influence on the model fit.

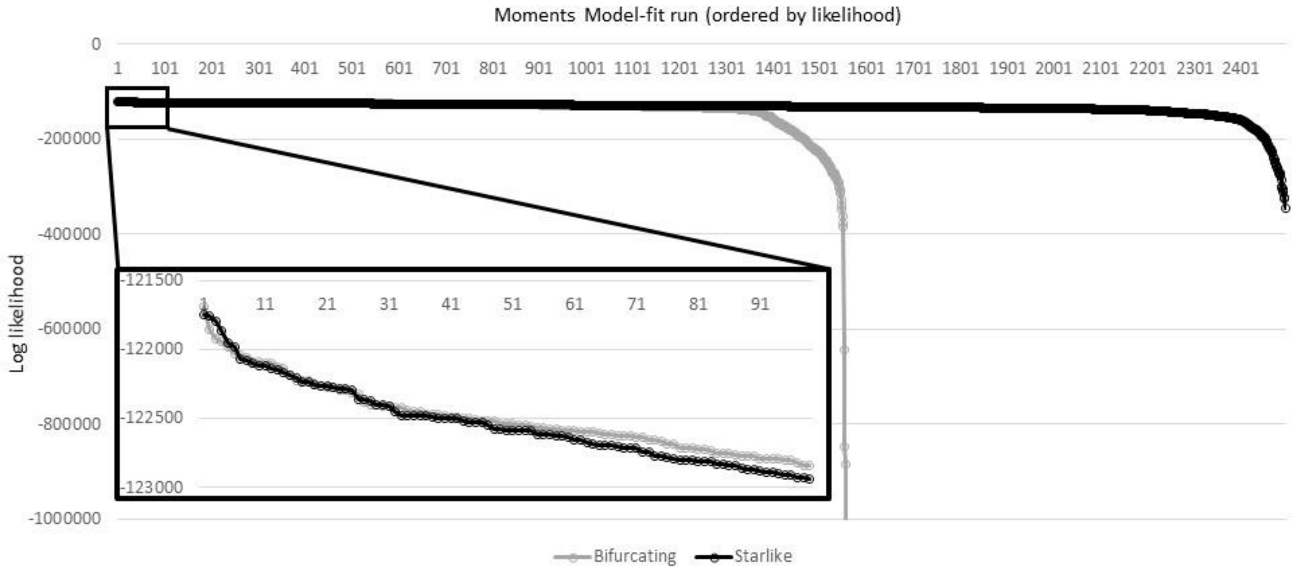

Figure S3: Model fit runs – loglikelihood, showing all runs (2,500), and the highest likelihood 100 runs (inset) for both four-population models

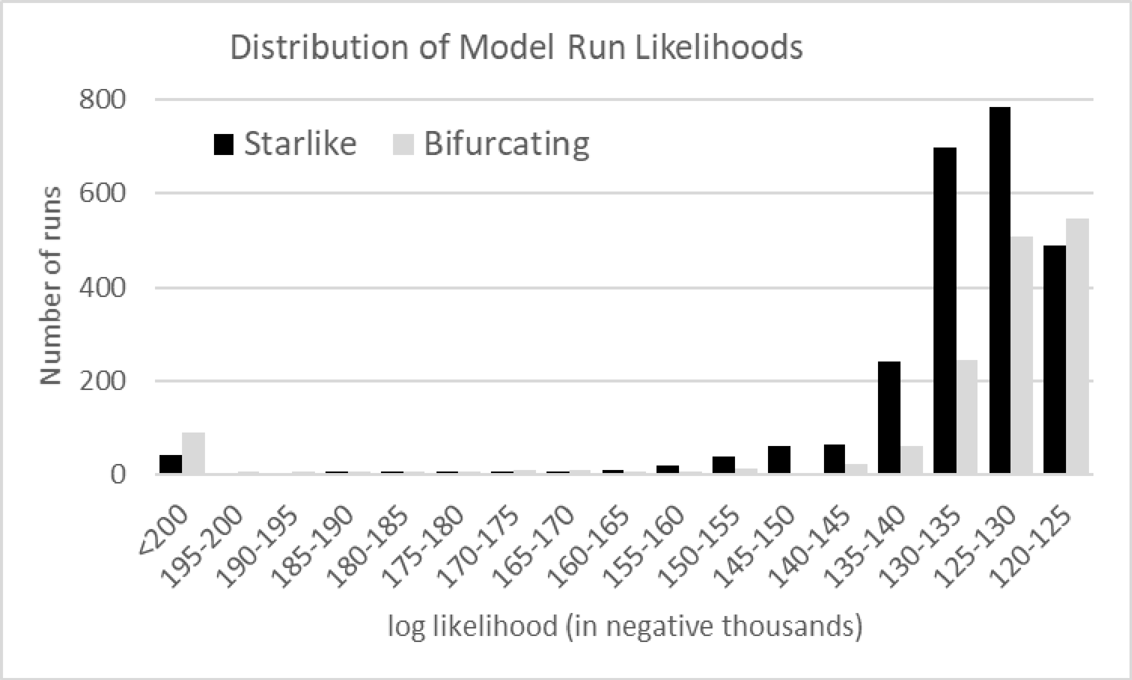

Figure S4: Likelihood distribution for moments model runs – both starlike and bifurcating models.

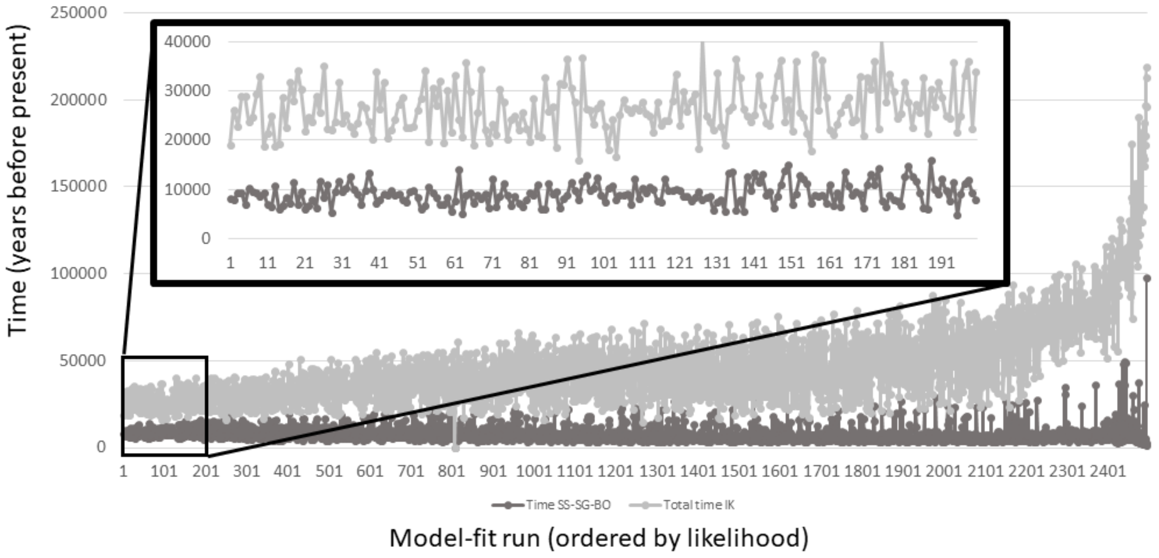

Figure S5: Model fit runs – population split timing – starlike model, showing all runs (2,500), and the highest likelihood 200 runs (inset). In dark grey – time (from present) to divergence of the South Shetlands, South Georgia, and Bouvetøya, in light grey – time (from present) to divergence of Iles Kerguelen

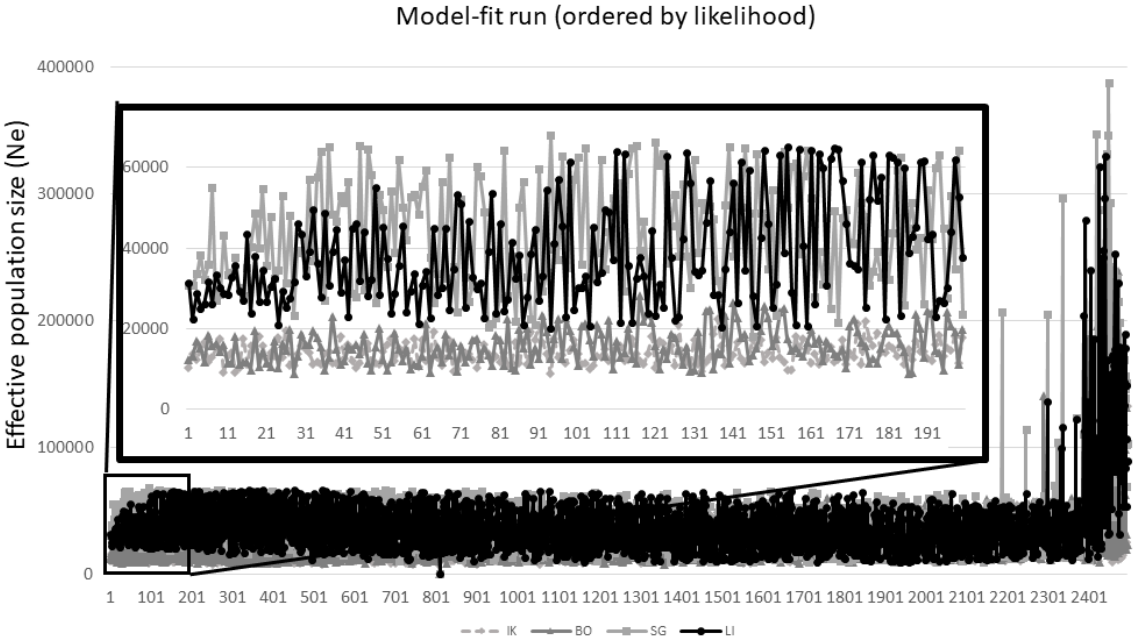

Figure S6: Model fit runs – population split sizes – starlike model, showing all runs (2,500), and the highest likelihood 200 runs (i SS

SS

Scripts

In the interests of brevity, where the same calculations were performed for each of the four island colonies, only the scripting for South Georgia is shown. Similarly, for all analyses multiple parameter sets were tested, but only one set is shown.

Site Frequency Spectrum generation in ANGSD

Bash script

```
#!/bin/bash
#Job name:
#SBATCH --job-name=SFS_for_demography
#
# Project:
#SBATCH --account=NN9515K
#
# Wall clock limit:
#SBATCH --time=12:00:00
#
#Max memory usage per core (MB):
#SBATCH --mem-per-cpu=5G
#
#Number of cores:
#SBATCH --cpus-per-task=5

## Set up job environment:
source /cluster/bin/jobsetup
module purge   # clear any inherited modules
set -o errexit # exit on errors

## make SFS files for each colony individually to use for demographic estimation

module load xz

for colony in LI SG BI KI
do
programs/angsd/angsd -bam AFS_ddRAD_analyses/"$colony"_AFS_bamlist.filelist -
doSaf 1 -out AFS_ddRAD_analyses/2019/"$colony"_AFS_SFS -anc
Reference_sequences/Seals/Arctocephalus_gazella.fasta -GL 2 -P 5 -minMapQ 1 -
minQ 20 -fold 1
```

programs/angsd/misc/realSFS AFS_ddRAD_analyses/2019/"$colony"_AFS_SFS.saf.idx > AFS_ddRAD_analyses/2019/"$colony"_AFS_SFS_complete
done

#Make a fourway SFS – note this was originally a separate script, as the Abel computing cluster was phased out, and we migrated to the new Saga computing cluster. Hence the slightly different file structure. Additionally in the fourway SFS, unfolded spectra were used as the folding format of ANGSD differs from that expected by moments. This was subsequently folded in moments, as recommended by the program authors.

Programs/ANGSD2/angsd/misc/realSFS AFS_ddRAD_analyses/2019/KI_AFS_unfolded.saf.idx AFS_ddRAD_analyses/2019/SG_AFS_unfolded.saf.idx AFS_ddRAD_analyses/2019/BI_AFS_unfolded.saf.idx AFS_ddRAD_analyses/2019/LI_AFS_unfolded.saf.idx> AFS_ddRAD_analyses/2019/IK_SG_BO_LI_4d_SFS_unfolded.sfs

Stairway

#South Georgia _demography blueprint file, longterm
#input setting
popid: SG_AFS_longterm_demography # id of the population (no white space)
nseq: 26 # number of sequences
L: 24157307 # total number of observed nucleic sites, including polymorphic and monomorphic
whether_folded: true # whether the SFS is folded (true or false)
SFS: 70137.94244 34262.07326 24407.76026 18495.96525 14527.33497 11965.19162 9956.172795 8728.44419 8342.75455 7223.10188 7167.079538 6587.423885 4869.011751
# snp frequency spectrum: number of singleton, number of doubleton, etc. (separated by white space)
#smallest_size_of_SFS_bin_used_for_estimation: 1
#largest_size_of_SFS_bin_used_for_estimation: 13 # default is nseq/2 for folded SFS
pct_training: 0.67 # percentage of sites for training
nrand: 6 12 20 24 # number of random break points for each try
project_dir: SG_AFS_longterm_demography # project directory
stairway_plot_dir: stairway_plot_es # directory to the stairway plot files
ninput: 200 # number of input files to be created for each estimation
#output setting
mu: 1.2e-8 # assumed mutation rate per site per generation
year_per_generation: 10 # assumed generation time (in years)
#plot setting
plot_title: SG_AFS_longterm_demography # title of the plot
xrange: 0.1,10000 # Time (1k year) range; format: xmin,xmax; "0,0" for default
yrange: 0,0 # Ne (1k individual) range; format: xmin,xmax; "0,0" for default
xspacing: 2 # X axis spacing
yspacing: 2 # Y axis spacing
fontsize: 12 # Font size

CubSFS

R-script

```
ObsSFS_AFS_LI_rounded<-c(63486, 38840, 27505, 19829, 15525, 13120, 10606,
10062,  9032,  8324,  7829,  7653,  5544)
ObsSFS_AFS_IK_rounded<-c(43437, 32390, 27861, 22196, 18960, 15105, 13907,
12122, 11176, 10159,  9891,  8927,  7738)
ObsSFS_AFS_BO_rounded<-c(46190, 30322, 21354, 17490, 14062, 10695, 10680,
7352,  7585,  9961,  5040,  2442,  9395)
ObsSFS_AFS_SG_rounded<- c(70138, 34262, 24408, 18496, 14527, 11965,  9956,
8728,  8343,  7223,  7167,  6587,  4869)
total.nb.sites_AFS_LI<- 25790417
total.nb.sites_AFS_SG<- 24157307
total.nb.sites_AFS_BO<- 23185013
total.nb.sites_AFS_IK<- 31037895
n.samples_AFS <- 26

require(CubSFS)
set.seed(98765)
#mutation rate
mu <- 1.2*10^(-8)
# generation time
genyr <- 10
TimeInYears <-seq(100,500000,by=100)
nb.boot<- 200

#South Shetlands
results_AFS_LI_optimal <- estimateCubSFS(ObsSFS_AFS_LI_rounded,
n.samples_AFS, n.knots=4,t_m=1.25, is.folded = TRUE)
boot_AFS_LI_optimal <-
rmultinom(nb.boot,total.nb.sites_AFS_LI,c(total.nb.sites_AFS_LI-
sum(ObsSFS_AFS_LI_rounded),ObsSFS_AFS_LI_rounded)/total.nb.sites_AFS_LI)
boot_AFS_LI_optimal<-boot_AFS_LI_optimal[-1,]
boot.res_AFS_LI_optimal<-apply(boot_AFS_LI_optimal, 2, estimateCubSFS,
n.samples=n.samples_AFS, n.knots=4, t_m=1.25, is.folded = TRUE)

AFS_plotting_LI_optimal <-
PlotCubSFS(n.samples_AFS,mu,total.nb.sites_AFS_LI,genyr,results_AFS_LI_optimal,
boot.res=boot.res_AFS_LI_optimal,abstime=TimeInYears)

#South Georgia
results_AFS_SG_optimal <- estimateCubSFS(ObsSFS_AFS_SG_rounded,
n.samples_AFS, n.knots=4,t_m=0.25, is.folded = TRUE)
```

```
boot_AFS_SG_optimal <-
rmultinom(nb.boot,total.nb.sites_AFS_SG,c(total.nb.sites_AFS_SG-
sum(ObsSFS_AFS_SG_rounded),ObsSFS_AFS_SG_rounded)/total.nb.sites_AFS_SG)
boot_AFS_SG_optimal<-boot_AFS_SG_optimal[-1,]
boot.res_AFS_SG_optimal<-apply(boot_AFS_SG_optimal, 2, estimateCubSFS,
n.samples=n.samples_AFS, n.knots=4, t_m=0.25, is.folded = TRUE)

AFS_plotting_SG_optimal <-
PlotCubSFS(n.samples_AFS,mu,total.nb.sites_AFS_SG,genyr,results_AFS_SG_optim
al,boot.res=boot.res_AFS_SG_optimal,abstime=TimeInYears)

#Bouvetøya
results_AFS_BO_optimal <- estimateCubSFS(ObsSFS_AFS_BO_rounded,
n.samples_AFS, n.knots=4,t_m=1.85, is.folded = TRUE)
boot_AFS_BO_optimal <-
rmultinom(nb.boot,total.nb.sites_AFS_BO,c(total.nb.sites_AFS_BO-
sum(ObsSFS_AFS_BO_rounded),ObsSFS_AFS_BO_rounded)/total.nb.sites_AFS_BO
)
boot_AFS_BO_optimal<-boot_AFS_BO_optimal[-1,]
boot.res_AFS_BO_optimal<-apply(boot_AFS_BO_optimal, 2, estimateCubSFS,
n.samples=n.samples_AFS, n.knots=4, t_m=1.85, is.folded = TRUE)

AFS_plotting_BO_optimal <-
PlotCubSFS(n.samples_AFS,mu,total.nb.sites_AFS_BO,genyr,results_AFS_BO_optim
al,boot.res=boot.res_AFS_BO_optimal,abstime=TimeInYears)

#Kerguelen
results_AFS_IK_optimal <- estimateCubSFS(ObsSFS_AFS_IK_rounded,
n.samples_AFS, n.knots=16,t_m=2, is.folded = TRUE)
boot_AFS_IK_optimal <-
rmultinom(nb.boot,total.nb.sites_AFS_IK,c(total.nb.sites_AFS_IK-
sum(ObsSFS_AFS_IK_rounded),ObsSFS_AFS_IK_rounded)/total.nb.sites_AFS_IK)
boot_AFS_IK_optimal<-boot_AFS_IK_optimal[-1,]
boot.res_AFS_IK_optimal<-apply(boot_AFS_IK_optimal, 2, estimateCubSFS,
n.samples=n.samples_AFS, n.knots=16, t_m=2, is.folded = TRUE)

AFS_plotting_IK_optimal <-
PlotCubSFS(n.samples_AFS,mu,total.nb.sites_AFS_IK,genyr,results_AFS_IK_optimal
,boot.res=boot.res_AFS_IK_optimal,abstime=TimeInYears)
```

Epos

Bash script – as formatted for the Saga computer cluster

```
#!/bin/bash
#
#Job name:
#SBATCH --job-name=epos
#
# Project:
#SBATCH --account=NN9515K
#
# Wall clock limit:
#SBATCH --time=24:00:00
#
#Max memory usage per core (MB):
#SBATCH --mem-per-cpu=5G
#
#Number of cores:
#SBATCH --cpus-per-task=1
#
#Send the output to the epos directory
#SBATCH --output=Programs/epos/LI_epos_complete_%j.out

## Set up job environment:
#source /cluster/bin/jobsetup
module purge   # clear any inherited modules
set -o errexit # exit on errors
set -o nounset # Treat unset variables as errors
module load foss/2019b GSL/2.6-GCC-8.3.0

Programs/sfs/build/bootSfs -i 2000 Programs/epos/data/LI_SFS_for_EPOS |
Programs/epos/build/epos -u 1.2e-8 -E 26 | ~/go/bin/epos2plot >
Programs/epos/results/LI_epos_results_2000bs_complete.dat
```

Moments

All moments scripts are in Python, and are accompanied by a shell script which is in bash, to allow implementation of the python scripts in the Unix environment.

Scenario definitions

```
def slightly_fancy_split(params, ns):
#Make a very simple scenario to estimate time of the split between two populations - before, during, or after LGM
#Assume both populations are of constant size - but may be of different sizes from each other
#params = time - time since populations split (in coalescent units), f = fraction of initial population which goes to pop 1 after splitting
#input SFS in order pop2, pop 1
#ns=[n0, n1 ] - sample sizes

    time, f = params
    sts = moments.LinearSystem_1D.steady_state_1D(ns[0] + ns[1])
    fs = moments.Spectrum(sts)
    fs = moments.Manips.split_1D_to_2D (fs, ns[0], ns[1])
    fs.integrate([f, (1-f)], time)
    return fs

def four_AFS_populations (params, ns):
#Make a very very simple scenario to estimate time of the split between each of the four distinct populations
#Assume all pops of  constant size
#populations are allowed to each be of different size
#params = T1 - time since SS-SG split, T2 - time between SS-SG split & SS/SG - BO split, T3 - time between SS/SG - BO split & SS/SG/BO - IK split, when each island splits off it gets its own population size
#input SFS in order IK, BO, SG, SS
#ns=[n0, n1, n2, n3] - sample sizes
#
    T1, T2, T3, IK, BO, SG, LI = params
    sts = moments.LinearSystem_1D.steady_state_1D(ns[0] + ns[1] + ns[2] + 
ns[3])
    fs = moments.Spectrum(sts)
    fs = moments.Manips.split_1D_to_2D (fs, ns[0], (ns[1] + ns[2]+ ns[3]))
    fs.integrate([IK, (BO + SG+ LI)], T3)
    fs = moments.Manips.split_2D_to_3D_2 (fs, ns[1], (ns[2] + ns[3]))
    fs.integrate([IK, BO, (SG + LI)], T2)
    fs = moments.Manips.split_3D_to_4D_3 (fs, ns[2], ns[3])
    fs.integrate([IK, BO, SG, LI], T1)
    return fs
```

```
	def four_AFS_pops_starlike (params, ns):
#A scenario in which the three "western" pops all divide simultaneously, with IK dividing out earlier
#populations are allowed to each be of different size
#params = T1 - time since SS-SG-BO split, T3 - time between SS/SG/BO split & SS/SG/BO - IK split, when each island splits off it gets its own population size (there is no time 2 - this is to maintain same parameter names as other scenarios)
#input SFS in order IK, BO, SG, SS
#ns=[n0, n1, n2, n3] - sample sizes

	T1, T3, IK, BO, SG, LI = params
	sts = moments.LinearSystem_1D.steady_state_1D(ns[0] + ns[1] + ns[2] + ns[3])
	fs = moments.Spectrum(sts)
	fs = moments.Manips.split_1D_to_2D (fs, ns[0], (ns[1] + ns[2]+ ns[3]))
	fs.integrate([IK, (BO + SG+ LI)], T3)
	fs = moments.Manips.split_2D_to_3D_2 (fs, ns[1], (ns[2] + ns[3]))
	fs.integrate([IK, BO, (SG + LI)], 0)
	fs = moments.Manips.split_3D_to_4D_3 (fs, ns[2], ns[3])
	fs.integrate([IK, BO, SG, LI], T1)
	return fs

def four_AFS_pops_star_with_migration (params, ns):
#A scenario in which the three "western" pops all divide simultaneously, with IK dividing out earlier
#After splitting, SG & LI are allowed symetric migration - to explain their closer relatedness as compared to BO
#populations are allowed to each be of different size - because SG is currently so much larger than the others this seems like a reasonable addition to make
#params = T1 - time since SS-SG-BO split, T3 - time between SS/SG/BO split & SS/SG/BO - IK split, when each island splits off it gets its own population size (there is no time 2 - this is to maintain same parameter names as other scenarios)
#m is migration between SG and LI (in fraction of source population*2Ne)
#input SFS in order IK, BO, SG, SS
#ns=[n0, n1, n2, n3] - sample sizes

	T1, T3, IK, BO, SG, LI, m = params
	sts = moments.LinearSystem_1D.steady_state_1D(ns[0] + ns[1] + ns[2] + ns[3])
	fs = moments.Spectrum(sts)
	fs = moments.Manips.split_1D_to_2D (fs, ns[0], (ns[1] + ns[2]+ ns[3]))
	fs.integrate([IK, (BO + SG+ LI)], T3)
	fs = moments.Manips.split_2D_to_3D_2 (fs, ns[1], (ns[2] + ns[3]))
```

```
    fs.integrate([IK, BO, (SG + LI)], 0)
    fs = moments.Manips.split_3D_to_4D_3 (fs, ns[2], ns[3])
    fs.integrate([IK, BO, SG, LI], T1, m = numpy.array([[0, 0, 0, 0], [0, 0, 0, 0],[0, 0, 0, m], [0, 0, m, 0]]))
    return fs
```

Model fitting

```
print('very begining of python script')
import matplotlib
matplotlib.use('Agg') # this should let me make plots without seeing them - e.g. on abel/saga
import matplotlib.pyplot as plt
import numpy
import cython
import mpmath
import networkx
import pandas
import moments
import AFS_demographic_scenarios_16
print('stuff imported - AFS model fitting 9')

unfolded_data_colony=moments . Spectrum . from_file('IK_BO_SG_LI_4d_SFS_unfolded.sfs')
data=unfolded_data_colony.fold()
ns = data.sample_sizes
func = AFS_demographic_scenarios_16.four_AFS_populations
#params are T1, T2, T3, IK, BO, SG, LI
upper_bound = [1, 1, 1, 10, 10, 10, 10]
lower_bound = [0.0001, 0.0001, 0.0001, 0.01, 0.01, 0.01, 0.01]
p0 = [0.5, 0.5, 0.5, 0.25, 0.25, 0.25, 0.25]

## Perturb our parameters before optimization. This does so by taking each
## parameter a up to a factor of two up or down.
p0 = moments.Misc.perturb_params(p0, fold=1, upper_bound=upper_bound, lower_bound=lower_bound)

popt = moments.Inference.optimize_log(p0, data, func, lower_bound=lower_bound, upper_bound=upper_bound, verbose=(len(p0)), multinom=False, maxiter=1000)

### Calculate the best-fit model AFS.
model = func(popt, ns)
### Likelihood of the data given the model AFS.
ll_model = moments.Inference.ll_multinom(model, data)
theta = moments.Inference.optimal_sfs_scaling(model, data)
print('fourway_split_model_IK_BO_SG_LI Optimal value of theta : {0}'.format(theta))
print('fourway_split_model_IK_BO_SG_LI Best-fit parameters : {0}'.format(popt))
print('fourway_split_model_IK_BO_SG_LI Maximum log composite likelihood : {0}'.format(ll_model))
```

Model fitting shell

```
#!/bin/bash
#Job name:
#SBATCH --job-name=4d_split
#
# Project:
#SBATCH --account=NN9515K
#
# Wall clock limit:
#SBATCH --time=12:00:00
#
#Max memory usage per core (MB):
#SBATCH --mem-per-cpu=5G
#
#Number of cores:
#SBATCH --cpus-per-task=1
#
# another 500 jobs will run in this array at the same time
#SBATCH --array=1-500
#
#Send the output to the moments directory
#SBATCH --
output=Programs/Moments/Output/fourway_starlike_model_IK_BO_SG_LI_fitting_
%j.out

## Set up job environment:
#source /cluster/bin/jobsetup
module purge   # clear any inherited modules
set -o errexit # exit on errors
set -o nounset # Treat unset variables as errors

module load Python/3.7.4-GCCcore-8.3.0
module load SciPy-bundle/2019.10-intel-2019b-Python-3.7.4
module list

cd Programs/Moments/moments_2020/

# each job will see a different ${SLURM_ARRAY_TASK_ID}
echo "now processing task id:: " ${SLURM_ARRAY_TASK_ID}
python -u AFS_four_way_model_fitting_9.py >
../Output/fourway_split_model_IK_BO_SG_LI_fitting_array_${SLURM_ARRAY_TAS
K_ID}.txt
```

Illustration

```
#A script to plot a spiffy figure of population history
import matplotlib
matplotlib.use('Agg') # to make compatible with the Abel/Saga computing clusters
import matplotlib.pyplot as plt
import numpy
import moments
import AFS_demographic_scenarios_18

#starlike model
func = AFS_demographic_scenarios_18.four_AFS_pops_starlike
popt = [0.00593865, 0.00803673, 0.15200561, 0.18012004, 0.4511215, 0.45885403]
ns = [27, 27, 27, 27]
model = moments.ModelPlot.generate_model(func, popt, ns)
moments.ModelPlot.plot_model(model, save_file='Starlike_4_way_bestfit_model.jpg',
pop_labels= ['IK', 'BØ', 'SG', 'LI'], fig_title='Best-fit Starlike Model', nref=1,
gen_time=10, gen_time_units='Years', reverse_timeline=True, draw_scale=0,
pop_color='DarkSlateBlue')

#Bifurcating model
func = AFS_demographic_scenarios_18.four_AFS_populations
popt = [0.00526866, 0.0009508, 0.01053675, 0.17880176, 0.18352939, 0.43806924,
0.42867754]
ns = [27, 27, 27, 27]
model = moments.ModelPlot.generate_model(func, popt, ns)
moments.ModelPlot.plot_model(model,
save_file='Bifurcating_4_way_bestfit_model.jpg', pop_labels= ['IK', 'BØ', 'SG', 'LI'],
fig_title='Best-fit Bifurcating Model', nref=1, gen_time=10, gen_time_units='Years',
reverse_timeline=True, draw_scale=0, pop_color='LightSeaGreen')
```

Site Frequency spectra for single colonies

South Shetlands
25553057.799840 63487.467737 38842.370006 27502.246436 19827.536880 15546.198121 13100.330814 10604.761716 10049.299586 9085.431885 8289.342069 7790.740682 7729.555159 5503.919067

South Georgia
23930656.602436 70136.047269 34260.225672 24403.499580 18505.560092 14494.259381 12015.242932 9905.691773 8731.287584 8415.540759 7071.762256 7358.078227 6388.024432 4965.177606

Bouvetøya
22992438.330913 46189.660833 30409.390996 21224.213546 17558.355220 13691.143159 12048.080765 7829.214719 11279.476092 4483.580895 10857.532247 5225.604281 3083.901721 8694.514613

Illes Kerguelen
30804020.103347 43428.667162 32448.283605 27775.347198 22262.607846 18949.202816 15084.200371 13962.839328 12001.741571 11269.094011 10052.381619 9930.139917 9004.795216 7705.595993
